# Single cell RNA-seq analysis identifies junctional adhesion molecule two positive endothelial cells aggravate osteoarthritis by mediating vascular permeability and macrophage adhesion

**DOI:** 10.3389/fcell.2026.1937741

**Published:** 2026-10-09

**Authors:** Yang Xing, Chengxin Li, Chao Wang, Linli Zheng, Puyi Sheng

**Affiliations:** 1 Department of Joint Surgery, The First Affiliated Hospital of Sun Yat-Sen University, Guangzhou, Guangdong, China; 2 Department of Orthopaedics, South China Hospital, Medical School, Shenzhen University, Shenzhen, Guangdong, China

**Keywords:** endothelial cells, junctional adhesion molecule 2, macrophage adhesion, osteoarthritis, single-cell RNA sequencing, vascular permeability

## Abstract

**Background:**

Osteoarthritis (OA) is a degenerative joint disease characterized by chronic inflammation and progressive cartilage destruction. Endothelial cell (EC) dysfunction contributes to inflammatory amplification in OA through increased vascular permeability and immune cell recruitment. This study aimed to characterize EC heterogeneity in OA and investigate the role of junctional adhesion molecule 2 (JAM2)-positive ECs in vascular dysfunction and macrophage adhesion.

**Methods:**

Single-cell RNA sequencing data from the Gene Expression Omnibus database were analyzed to identify EC subpopulations in OA. JAM2 expression was validated in human OA synovium, meniscus, and subchondral bone using multiplex immunofluorescence staining. The effects of inflammatory cytokines, including TNF-α, IL-1β, IL-6, and TGF-β1, on JAM2 expression in ECs were evaluated by qRT-PCR and immunofluorescence. The functional effects of JAM2 blockade on EC migration, tube formation, endothelial permeability, and monocyte adhesion were assessed using *in vitro* assays. The therapeutic potential of JAM2 blockade was further evaluated in a destabilization of the medial meniscus (DMM)-induced mouse model of OA.

**Results:**

JAM2-positive ECs were enriched in pathways associated with cell adhesion and transendothelial migration. JAM2 expression was significantly increased in OA synovial tissue compared with controls. Among the inflammatory cytokines tested, TNF-α most strongly upregulated JAM2 expression in ECs. TNF-α stimulation impaired EC migration and tube formation, increased endothelial permeability, and enhanced monocyte adhesion, whereas JAM2 blockade partially attenuated these effects *in vitro*. In the DMM-induced OA model, JAM2 expression was increased in the vascular compartments of the synovium, meniscus, and subchondral bone, while JAM2 blockade attenuated cartilage degeneration and modulated macrophage infiltration.

**Conclusion:**

JAM2-positive ECs contribute to OA progression by promoting vascular dysfunction and macrophage adhesion. Targeting JAM2-positive ECs may represent a potential therapeutic strategy for alleviating chronic inflammation in OA.

## Introduction

Osteoarthritis (OA) is a prevalent form of arthritis, primarily affecting weight-bearing joints such as the knees, hips, and interphalangeal joints of the fingers (Martel-Pelletier et al., 2016). In its advanced stages, OA leads to severe pain, disability, and significant healthcare and socioeconomic burdens. The Global Burden of Disease (GBD) estimates indicate that over 28% of older adults suffer from OA (Long et al., 2022), with the number of affected individuals projected to rise due to population aging and longer life expectancy (Safiri et al., 2021). Despite its high prevalence, the underlying mechanisms of OA remain incompletely understood (Yao et al., 2023).

Traditionally, OA has been classified as an age-related degenerative disorder, characterized by progressive degradation and destruction of articular cartilage (Pritzker et al., 2006). However, growing evidence suggests that OA is a chronic inflammatory disease involving the entire joint. Persistent inflammation can lead to synovial hyperplasia, inflammatory cell infiltration, and osteophyte formation, creating a self-perpetuating cycle of joint inflammation that drives OA progression (Motta et al., 2023; Sanchez-Lopez et al., 2022; Zhang et al., 2019).

Recent studies have highlighted a critical role for angiogenesis and endothelial cell (EC) dysfunction in OA, where aberrant EC behavior may exacerbate synovial inflammation and cartilage degeneration (Jeong et al., 2021; Su et al., 2020). During angiogenesis, ECs undergo phenotypic changes—including loss of barrier function, extracellular matrix degradation, altered adhesive properties, migration, and tube formation—that promote new blood vessel formation and potentially facilitate inflammatory cell infiltration in OA. Vascular integrity and transendothelial migration (TEM) are tightly regulated by EC adhesion molecules such as platelet endothelial cell adhesion molecule (PECAM), intercellular adhesion molecule (ICAM), endothelial cell-selective adhesion molecule (ESAM), and junctional adhesion molecules (JAMs) (Rohlenova et al., 2020; Lee et al., 2021; Schimmel and Gordon, 2018), which may modulate immune cell trafficking into the synovium. Despite these insights, the contribution of EC-mediated processes to OA pathogenesis remains poorly understood.

In this study, we aimed to identify OA-specific EC subpopulations and uncover potential therapeutic targets. We integrated single-cell RNA sequencing (scRNA-seq) data from OA knee synovium, meniscus, and subchondral bone to map the EC landscape and characterize transcriptional heterogeneity. This analysis revealed an adhesion-related EC subcluster and identified junctional adhesion molecule 2 (JAM2) as a candidate marker. Our findings indicate that JAM2-positive ECs may play a critical role in OA pathophysiology, providing a basis for future diagnostic and therapeutic investigations.

## Materials and methods

### Collection of scRNA-seq data

We searched for single-cell RNA sequencing (scRNA-seq) studies of human osteoarthritis (OA) in the Gene Expression Omnibus (GEO) repository. Publicly available datasets from GSE152805, GSE196678, and GSE220243 were downloaded. The GSE152805 dataset includes nine samples, consisting of three osteoarthritic synovial membranes and six osteoarthritic cartilage samples. GSE196678 contains four subchondral bone samples from two OA patients. The GSE220243 dataset includes seven meniscus samples from normal joints (one avascular sample) and six meniscus samples from osteoarthritic joints. For the purpose of ensuring an adequate number of endothelial cells (ECs) for analysis, data from cartilage and avascular meniscus were excluded after preliminary evaluation. All data analyses were conducted using R software (version 4.3.1; https://www.r-project.org/). A graphical flowchart summarizing the study process is presented in Figure 1.

**FIGURE 1:**
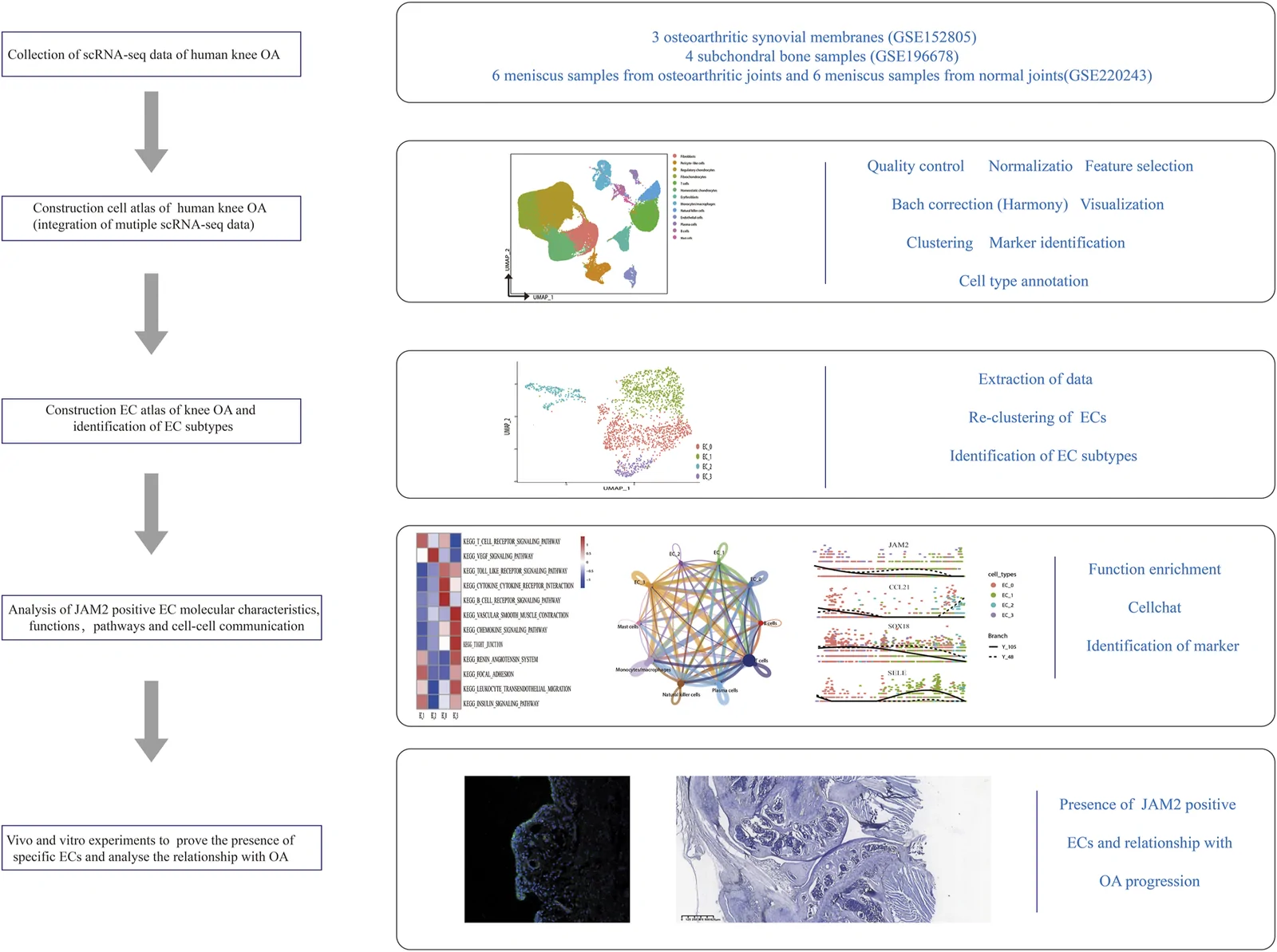
Workflow of the integrated analysis in this study.

### Integration and analysis of multi-tissue scRNA-seq datasets from knee osteoarthritis

To improve statistical power and facilitate the identification of shared cellular programs across anatomically related joint tissues, we integrated scRNA-seq data from three publicly available knee OA datasets. All datasets were generated from OA patients using comparable droplet-based sequencing platforms, which allowed for cross-dataset integration with minimal technical bias. Data integration was performed to mitigate batch effects, ensuring biological variability was preserved across the tissues.

Gene expression matrices were imported using the Read10X function and processed to generate Seurat objects (Seurat v4.0.5). Quality control steps were applied to each dataset to exclude low-quality cells and potential artifacts. Specifically, cells with fewer than 2,000 detected genes, over 10,000 detected genes, or mitochondrial transcript proportions exceeding 10% were removed. After filtering, Seurat objects were merged into a single object using the merge function. Doublets were identified and removed with the DoubletFinder tool to reduce bias from multiplet events.

The merged dataset was normalized and scaled using Seurat’s NormalizeData and ScaleData functions. Highly variable genes were identified using the FindVariableFeatures function, and the top 2,000 most variable features were selected for downstream analysis. Principal component analysis (PCA) was performed using the RunPCA function to reduce dimensionality and capture primary sources of variation.

To correct for inter-dataset technical variability, batch effects were mitigated using the Harmony algorithm (Harmony v0.1.0) applied to the PCA embeddings via RunHarmony. Dataset identity was included as the batch covariate, while tissue identity was excluded to avoid over-correction of tissue-specific transcriptional signatures. The default Harmony parameters were applied for all settings.

Graph-based clustering was performed using FindNeighbors and FindClusters with the first 30 principal components and a resolution of 0.2. Uniform manifold approximation and projection (UMAP) was used for two-dimensional visualization of the cellular landscape. Differentially expressed genes for each cluster were identified using FindAllMarkers with an adjusted p-value <0.05, only. pos = TRUE, and logfc. threshold = 0.25. Cluster identities were assigned based on canonical marker genes from previous single-cell studies of joint tissues.

### Analysis of the EC atlas

To further investigate endothelial cells (ECs), ECs were subset from the scRNA-seq dataset based on canonical EC markers (PECAM1 and VWF). These ECs were then integrated using the Harmony package to mitigate batch effects across samples. Four distinct EC subclusters were identified and visualized using UMAP. To assess the functional activities of these subclusters, single-sample gene set enrichment analysis (ssGSEA) was performed using the GSVA package (v1.40.1). SsGSEA scores were calculated for Gene Ontology (GO) gene sets and the 50 Hallmark gene sets from the Molecular Signatures Database (MSigDB), allowing for characterization and comparison of pathway-level activities across clusters.

### Trajectory analysis

To explore lineage transitions and infer developmental trajectories across cell types, we used both Monocle 2, CytoTrace and Singleshot. A CellDataSet object was created from the UMI count matrix using the newCellDataSet function. Size factors and dispersions were estimated using estimateSizeFactors and estimateDispersions to normalize mRNA capture efficiency across cells and to support differential expression analysis. Ordering genes were selected using the differentialGeneTest function, applying a significance threshold of P < 0.01, and genes were required to be expressed in at least 10% of cells. These genes were used to infer the developmental trajectory. Dimensionality reduction was performed using reduceDimension (2 components), and cells were ordered along the inferred pseudotime trajectory. Pseudotime-dependent genes were identified using differentialGeneTest, and expression dynamics were visualized with plot_pseudotime_heatmap. CytoTrace was applied to estimate the differentiation potential and infer trajectory directionality. Cell distributions across pseudotime were visualized using the ggridges package (v0.5.3).

### Cell–cell interaction analysis

CellChat package (v2.2.0) was used to analyze cell–cell interactions between annotated cell types. The raw UMI count matrix exported from Seurat was paired with a corresponding metadata file containing cell type annotations for statistical analysis. Significant ligand–receptor pairs were identified, and interaction patterns were visualized using heatmaps and dot-plots generated by the CellChat tool. Additionally, chord diagrams were created using the circlize package (v0.4.14) to display the interaction connectivity across cell types. The interaction strength of significant ligand–receptor pairs was visualized using the pheatmap package (v1.0.12).

### Mice and clinical samples

All animal experiments were approved by the Laboratory Animal Welfare and Ethics Committee of The First Affiliated Hospital of Sun Yat-sen University (Approval No. 2023094). A total of 12 male C57BL/6 mice (10 weeks old) were included in this study and allocated into four groups (n = 3 per group): sham surgery, DMM at 4 weeks, DMM at 8 weeks, and DMM at 8 weeks treated with JAM2-neutralizing antibodies. DMM surgery was performed to induce OA, as described previously (Glasson et al., 2007; Xu et al., 2005). Under isoflurane anesthesia, a medial menisco-tibial ligament cut was made in the right knee joint. A placebo surgery was performed on control mice, following the same steps without cutting the ligament. Mice were euthanized four or 8 weeks after the procedure. For anti-JAM2 treatment, mice undergoing DMM surgery received an anti-mouse JAM2 monoclonal antibody (R&D Systems, Catalog #MAB9881; clone 150,005) by intraperitoneal injection beginning 1 day after surgery and continuing for 8 weeks. The antibody was administered at a dose of 100 μg/mouse every 3 days. Control mice received the corresponding isotype control antibody according to the same dosing schedule.

Six human synovial tissue samples were obtained from OA patients undergoing total knee arthroplasty (TKA), and from patients who had amputation due to osteosarcoma. The baseline characteristics of the patients are summarized in Supplementary Table S1. The samples were divided into two groups: the OA group, which showed significant cartilage injury in the medial compartment of subchondral bone and meniscus, and the control group, with relatively intact cartilage coverage in the lateral compartment. All patients gave informed consent to use their clinical information for scientific research. The study was approved by the Ethics Committee of the First Affiliated Hospital of Sun Yat-sen University (Approval No. 2021673).

### Immunofluorescence and immunohistochemistry

Cell immunofluorescence staining:HUVECs were stimulated with TNF-α (10 ng/mL) for 24 h. After washing with phosphate-buffered saline (PBS), cells were fixed in 4% paraformaldehyde at 4 °C for 10 min, permeabilized with 1% Triton X-100 in PBS for 15 min, and blocked for 1 h at room temperature. Cells were incubated overnight at 4 °C with a primary rabbit anti-human JAM2 antibody (Affinity Biosciences; 1:200; DF2550). After three PBS washes, cells were incubated with secondary antibody (Thermo Fisher Scientific; 1:400; A11008) for 1 h. DAPI was used for counterstaining before imaging.

### Tissue immunohistochemistry and immunofluorescence

Paraffin-embedded tissue sections were deparaffinized and rehydrated, followed by antigen retrieval. Endogenous peroxidase activity was quenched with 3% H2O2, and sections were blocked with goat serum. Immunohistochemistry (IHC) was performed on mouse knee sections, and immunofluorescence (IF) on human synovial tissues. For human synovium IF, rabbit anti-human JAM2 antibody (Affinity Biosciences; 1:200; DF2550) and rat anti-human CD31 antibody (Santa Cruz; 1:200; SC-18916) were incubated overnight at 4 °C. Fluorescence-labeled secondary antibodies, including donkey anti-rabbit IgG (H + L) conjugated to Alexa Fluor 594 (Thermo Fisher Scientific; 1:500; A-21207) and donkey anti-rat IgG (H + L) conjugated to Alexa Fluor 488 (Thermo Fisher Scientific; 1:1,000; A-21208), were applied for 1 h. For mice synovium IF, rat anti-mouse F4/80 antibody (Affinity Biosciences; 1:200; DF2789), rabbit anti-mouse NOS2 antibody (Abcam; 1:100; ab15323), and rabbit anti-mouse CD206 antibody (Abcam; 1:200; ab64693) were incubated overnight at 4 °C. Fluorescence-labeled secondary antibodies, including donkey anti-rat IgG (H + L) conjugated to Alexa Fluor 488 (Thermo Fisher Scientific; 1:500; A-21208) and donkey anti-rabbit IgG (H + L) conjugated to Alexa Fluor 594 (Thermo Fisher Scientific; 1:1,000; A-21207), were applied for 1 h. For IHC, primary antibody against JAM2 (Affinity Biosciences; 1:200; DF2550) was incubated overnight at 4 °C, and immunoreactivity was detected using the SP Rabbit and Mouse HRP Kit (DAB) (CW Biotech, China). Sections were counterstained with hematoxylin.

### Image analysis

Images were acquired using a fluorescence microscope (IF) or a bright-field microscope (IHC). Fluorescence intensity in CD31-positive regions of interest (ROIs) was measured using mean fluorescence intensity (MFI) and normalized for statistical analysis. F4/80^+^ macrophages expressing NOS2 or CD206 were identified based on the co-localization of F4/80 with NOS2 or CD206 fluorescence signals, respectively. The numbers of NOS2^+^ and CD206^+^ macrophages were quantified in randomly selected fields of view, and the analyzed tissue area was measured using ImageJ software. The numbers of NOS2^+^ and CD206^+^ macrophages were normalized to the corresponding tissue area and expressed as cells/mm^2^. Three fields were analyzed for each section, and the mean value was used for statistical analysis. DAB staining was quantified as mean optical density (MOD), calculated as integrated optical density divided by the positively stained area (IOD/area). Five non-overlapping fields per specimen were analyzed.

### Safranin O/Fast Green staining

Mice knee joints were fixed in 4% paraformaldehyde, decalcified in EDTA, paraffin-embedded, and sectioned at 5 μm. Sections were deparaffinized and rehydrated, followed by staining with Weigert’s iron hematoxylin, Fast Green, and Safranin O according to the manufacturer’s instructions (MCE, HY-K0607). After dehydration and clearing, sections were mounted and imaged using a bright-field microscope. Cartilage degeneration was assessed using the OARSI scoring system.

### Osteoarthritis research society international (OARSI) scoring

Cartilage degeneration was evaluated on Safranin O/Fast Green-stained sections according to the OARSI histopathological scoring system. Cartilage lesions were graded on a scale of 0–6, with 0 representing normal, intact cartilage and six representing severe cartilage destruction. Higher scores indicated more severe cartilage degeneration. Scoring was performed in a blinded manner by an investigator who was unaware of the experimental groups.

### Cell culture and experiments

Human umbilical vein endothelial cells (HUVECs) and the THP-1 monocytic cell line were obtained from BHcell (Guangzhou, China). HUVECs were cultured in Endothelial Cell Medium (ECM; ScienCell Research Laboratories, Carlsbad, CA, United States) supplemented with 10% fetal bovine serum (FBS), 1% endothelial cell growth supplement (ECGS), penicillin (100 U/mL), and streptomycin (100 μg/mL). HUVECs were treated with TNF-α (10 ng/mL), IL-1β (10 ng/mL), IL-6 (10 ng/mL), TGF-β (5 ng/mL), or PBS (vehicle control) for 24 h. After treatment, cells were subjected to qRT-PCR and IF analyses. HUVECs were treated with PBS, TNF-α (10 ng/mL), anti-JAM2 antibody (1 μg/mL), or a combination of TNF-α and anti-JAM2 antibody for 24 h. Following treatment, HUVECs were subjected to tube formation, migration, cell adhesion and permeability. THP-1 cells were cultured in RPMI 1640 with 10% FBS.

### Functional assays

Tube formation, migration, cell adhesion, and permeability assays were performed to assess EC function. Tube formation was evaluated in Matrigel-coated wells. Migration was assessed using a scratch wound assay. Cell adhesion was quantified after THP-1 cells adhered to the HUVEC monolayer. Permeability was measured using Transwell inserts with TRITC-dextran.

### Quantitative real-time polymerase chain reaction (qRT-PCR) analysis

Total RNA was extracted using TRIzol reagent (Invitrogen). cDNA synthesis was performed with a reverse transcription kit (Toyobo). qRT-PCR was performed using SYBR Green Mix (Toyobo) on a LightCycler 480 II system (Roche). GAPDH was used as the endogenous reference gene, and relative mRNA expression levels were calculated using the 2^−ΔΔCT^ method.

### Statistical analysis

Data are presented as mean ± SD from at least three independent experiments. Statistical analyses were performed using GraphPad Prism (version 5.0). Two-group comparisons were performed using a two-tailed Student’s t-test, while comparisons among multiple groups were analyzed using one-way ANOVA followed by Tukey’s *post hoc* test. A P-value <0.05 was considered statistically significant.

## Results

### Integrated cell atlas of human knee OA

After performing quality control and filtering, a total of 100,087 cells from 18 human knee OA samples were retained for downstream analysis. These cells were integrated using the Harmony algorithm to correct for batch effects. Unsupervised clustering coupled with UMAP visualization identified 13 major cell populations, including regulatory chondrocytes (RegCs), fibrochondrocytes (FCs), homeostatic chondrocytes (HomCs), fibroblasts (Fbs), erythroid cells, pericyte-like cells, monocytes/macrophages, T Cells, natural killer (NK) cells, endothelial cells (ECs), plasma cells, B Cells, and mast cells (Figure 2A).

**FIGURE 2:**
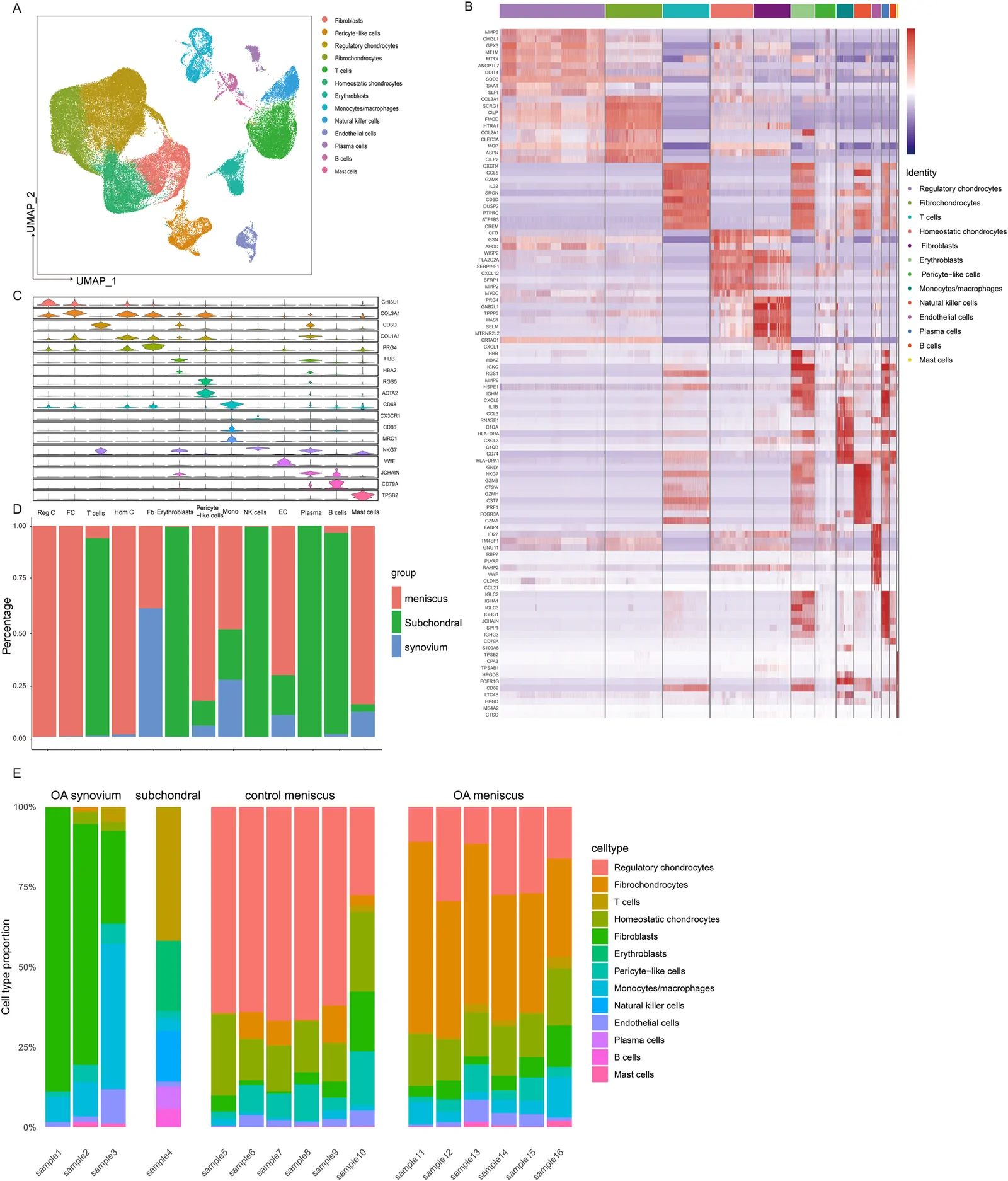
Single-cell RNA sequencing of human knee tissue. **(A)** UMAP plot of 100,087 cells showing unsupervised clusters of 13 identified main clusters in human knee OA tissue. **(B)** Heatmap revealing the scaled expression of differentially expressed genes for each cluster. **(C)** Violin plots showing the expression of the indicated markers for each cell cluster on the UMAP. **(D)** Bar plots showing the constituent ratio of each cluster in the three tissues. **(E)** Bar plots showing the relative abundance of major cell types across the 16 individual samples. Samples 1–3 were derived from synovial tissues of patients with OA, sample four represented a pooled subchondral bone dataset from four patients, including two patients with OA and two controls, samples 5–10 were derived from menisci of control patients, and samples 11–16 were derived from menisci of patients with OA.

Marker gene analysis revealed distinct transcriptional signatures across these clusters, as shown in a heatmap of the top differentially expressed marker genes (scaled expression values) for each population (Figure 2B). Representative genes included: CHI3L1 for RegCs, COL3A1 for FCs, COL1A1 for HomCs (Lv et al., 2022; Sun et al., 2020; Wang et al., 2021), CD3D for T Cells, HBB/HBA2 for erythroid cells, RGS5 and ACTA2 for pericyte-like cells, CX3CR1, CD86, CD206 and CD68 for monocytes/macrophages, NKG7 for NK cells, VWF for ECs, JCHAIN for plasma cells, CD79A for B Cells, and TPSB2 for mast cells (Chou et al., 2020) (Figure 2C).

Further analysis of cell-type composition revealed significant spatial heterogeneity across anatomical compartments. Immune and vascular-associated cell lineages were broadly distributed across all sampled sites, whereas chondrocyte-lineage and stromal populations showed site-specific enrichment (Figure 2D). To further characterize the cellular composition of individual samples, we examined the relative abundance of major cell types across all 16 samples (Figure 2E). Samples 1-3 were derived from synovium of patients with OA, sample four represented a pooled subchondral bone dataset generated from four patients (two with OA and two controls), samples 5-10 were derived from menisci of control patients, and samples 11-16 were derived from menisci of patients with OA. The cellular composition varied among individual samples, reflecting differences in anatomical origin and disease status (Figure 2E).

### Identification of EC characteristics in human OA knee

To characterize the features of ECs in OA, we extracted and reclustered ECs. In total, 2,089 ECs were classified into four distinct subclusters by unbiased clustering (Figure 3A). EC_0 predominantly expressed marker genes of venous endothelial cells, such as VWF and PLCG2 (Zhang et al., 2023). EC_1 expressed genes associated with cell growth, differentiation, and stress responses, including proto-oncogenes (JUNB, FOSB, FOS) and heat shock proteins (DNAJA1, DNAJB1), potentially indicating ECs in a proliferative phase (Alfonso-Jaume et al., 2006). EC_2 was identified as lymphatic endothelial cells, specifically expressing LYVE1 (Fujimoto et al., 2020). EC_3 represented an inflammation-associated cluster, with preferential expression of inflammation-related genes, including CXCL12, IGFBP3, JAG1, and FN1 (Figures 3B,D).

**FIGURE 3:**
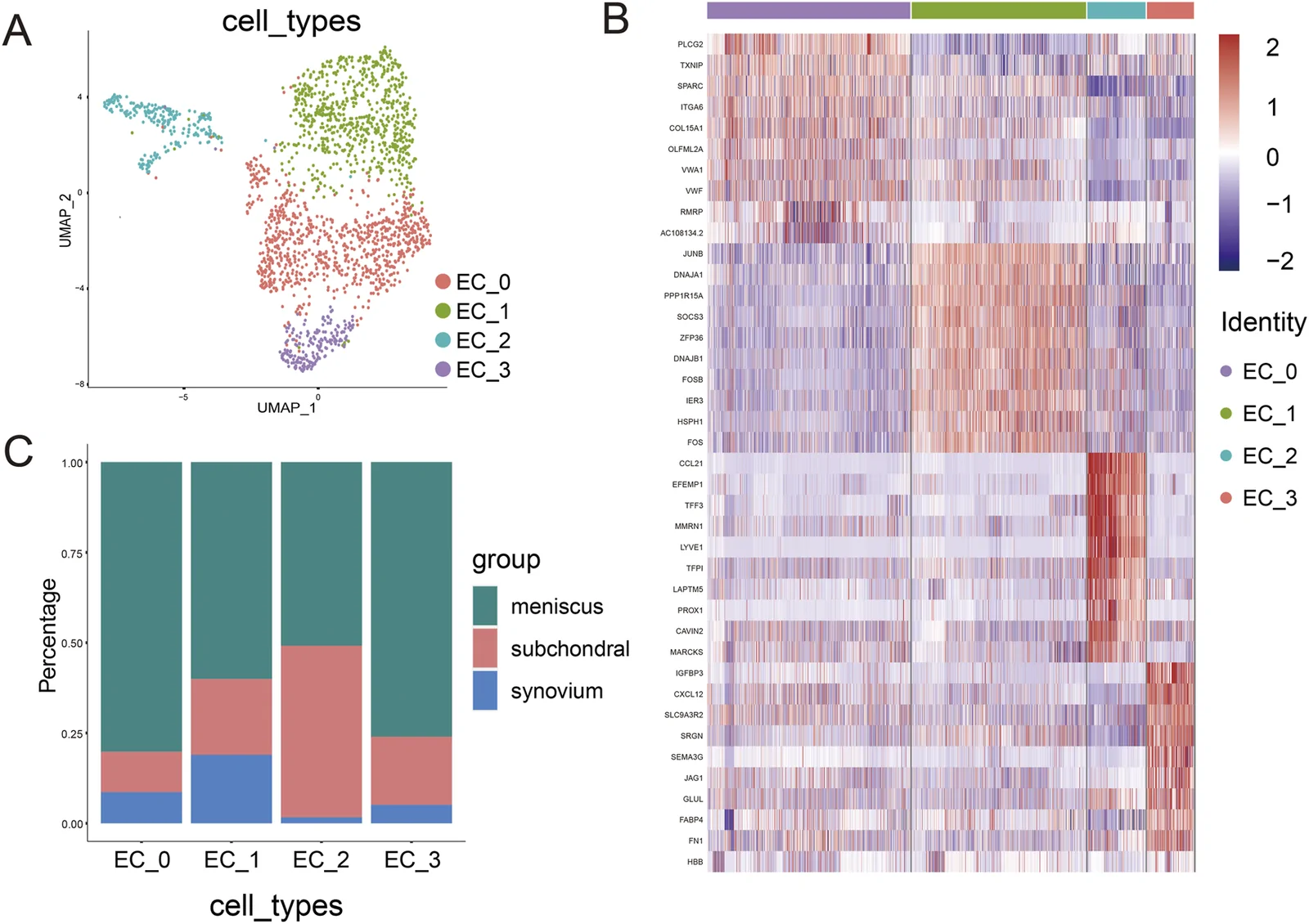
Integrated analysis of scRNA-seq data reveals the differences in EC composition of human knee tissue. **(A)** UMAP visualization of different EC subclusters from human knee tissue. **(B)** Heatmap to show the top DEGs (Wilcoxon test) in each EC subcluster. **(C)** Bar plots to show the proportion of different EC subclusters in each tissue.

We performed further analysis of the EC subclusters by randomly sampling 200 cells from each cluster and calculating the proportion of cell sources. We found that lymphatic ECs (EC_2) were predominantly derived from the subchondral bone, while other EC subtypes were relatively evenly distributed across the three tissues (Figure 3C). This suggests that each EC subcluster shares a common lineage rather than being restricted to a specific anatomical location. These results indicate that ECs play a significant role in the pathogenesis of OA, highlighting the importance of understanding their heterogeneity.

### EC_3 activated subcluster regulating adhesion and vascular permeability in OA

To further investigate the role of EC_3 in OA chronic inflammation, we conducted Gene Set Variation Analysis (GSVA) to uncover the biological characteristics of the four EC subclusters. Our analysis revealed that the B cell receptor signaling pathway and cytokine-cytokine receptor interaction KEGG terms were significantly enriched in EC_0, while T cell receptor signaling pathways were predominantly enriched in EC_1. In contrast, EC_3 exhibited enrichment in pathways related to vascular morphology and transendothelial migration (TEM) regulation, including tight junctions, the renin-angiotensin system, chemokine signaling, and leukocyte TEM (Figure 4A).

**FIGURE 4:**
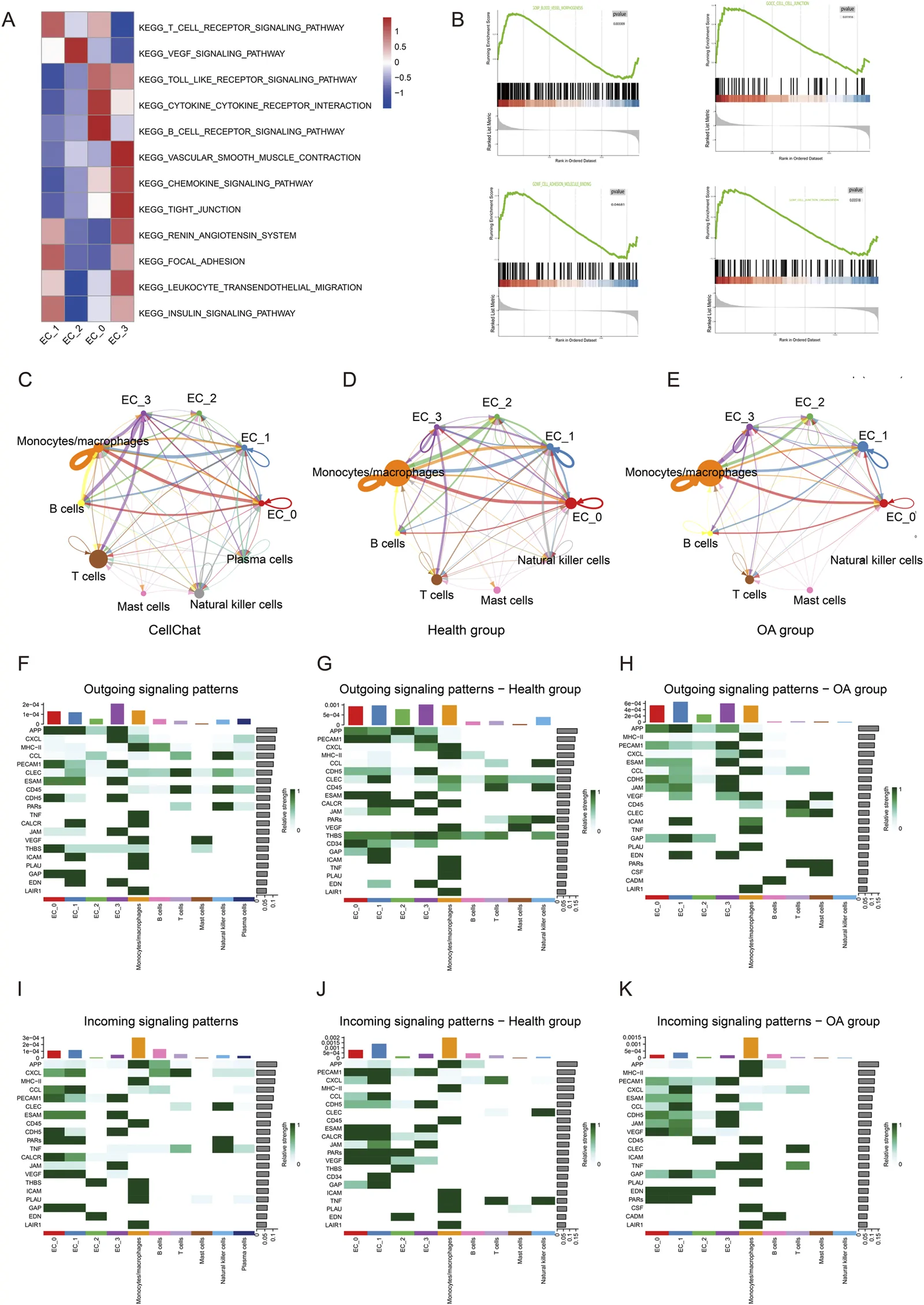
Identification and molecular characterization of the EC subcluster in human knee tissue. **(A)** Heatmap to show the differentially enriched KEGG terms in each EC subcluster. **(B)** GSEA revealing the signaling pathways enriched in EC_3 compared with other EC subclusters. **(C)** Overview of the cell-cell crosstalk and the communications between EC subclusters and immune cell populations. **(D)** Cellular communication network showing the predicted interactions between EC subclusters and immune cell populations in the healthy control group, consisting of meniscus samples from samples 5-10. **(E)** Cellular communication network showing the predicted interactions between EC subclusters and immune cell populations in the OA group, consisting of synovial samples from samples 1-3 and meniscus samples from samples 11-16. **(F)** Heatmap showing the relative strength of outgoing signaling pathways across EC subclusters and immune cell populations in the overall dataset. **(G)** Heatmap showing the relative strength of outgoing signaling pathways across EC subclusters and immune cell populations in the healthy group. **(H)** Heatmap showing the relative strength of outgoing signaling pathways across EC subclusters and immune cell populations in the OA group. **(I)** Heatmap showing the relative strength of incoming signaling pathways across EC subclusters and immune cell populations in the overall dataset. **(J)** Heatmap showing the relative strength of incoming signaling pathways across EC subclusters and immune cell populations in the healthy group. **(K)** Heatmap showing the relative strength of incoming signaling pathways across EC subclusters and immune cell populations in the OA group.

Gene Ontology (GO) analysis using Gene Set Enrichment Analysis (GSEA) further indicated that EC_3 was enriched in gene sets related to blood vessel morphogenesis, cell-cell junctions, cell adhesion molecule binding, and cell junction organization (Figure 4B). These findings suggest that EC_3 may facilitate OA progression by regulating vascular permeability and inflammatory cell TEM.

We next performed CellChat analysis to investigate the potential interactions between EC subclusters and immune cell populations. The cellular communication network showed that EC_3 interacted with multiple immune cell populations, including monocytes/macrophages, B cells, and T cells (Figure 4C). To further determine whether these interactions were altered in OA, we compared cellular communication between the healthy control group, consisting of meniscus samples from samples 5-10, and the OA group, consisting of synovium from samples 1-3 and meniscus samples from samples 11-16. Notably, EC_3 exhibited stronger predicted interactions with monocytes/macrophages in the OA group than in the healthy control group (Figures 4D,E). These findings suggest that EC_3 may participate in enhanced endothelial-myeloid cell communication in the OA microenvironment. We next examined the signaling pathways associated with EC_3 using CellChat analysis. The top enriched signaling pathways in the incoming and outgoing networks of EC_3 included PECAM, ESAM, CDH5, and JAM in both the healthy control and OA groups (Figures 4F–K). Notably, although these endothelial-associated signaling pathways were detected in both groups, the JAM signaling pathway showed markedly stronger activity in EC_3 in the OA group than in the healthy control group, in both incoming and outgoing signaling networks (Figures 4G,H,j,K). These findings suggest that JAM-mediated cellular communication involving EC_3 is enhanced in the OA microenvironment. Collectively, EC_3 represents an activated EC subcluster that may promote chronic inflammation in OA through regulating cell adhesion and vascular permeability.

### JAM2 is a potential marker of EC_3

The above findings suggest that EC_3 may play a key role in OA progression. To explore the differentiation trajectories of EC subclusters, we applied CytoTRACE, Monocle and SingleShot to establish a pseudotemporal order among cell types. Our analysis identified four major EC subclusters and revealed differences in their inferred differentiation potential. CytoTRACE analysis showed that EC_1 exhibited the lowest differentiation state, followed by EC_0, whereas EC_2 and EC_3 displayed higher differentiation potential (Figures 5A–C).

**FIGURE 5:**
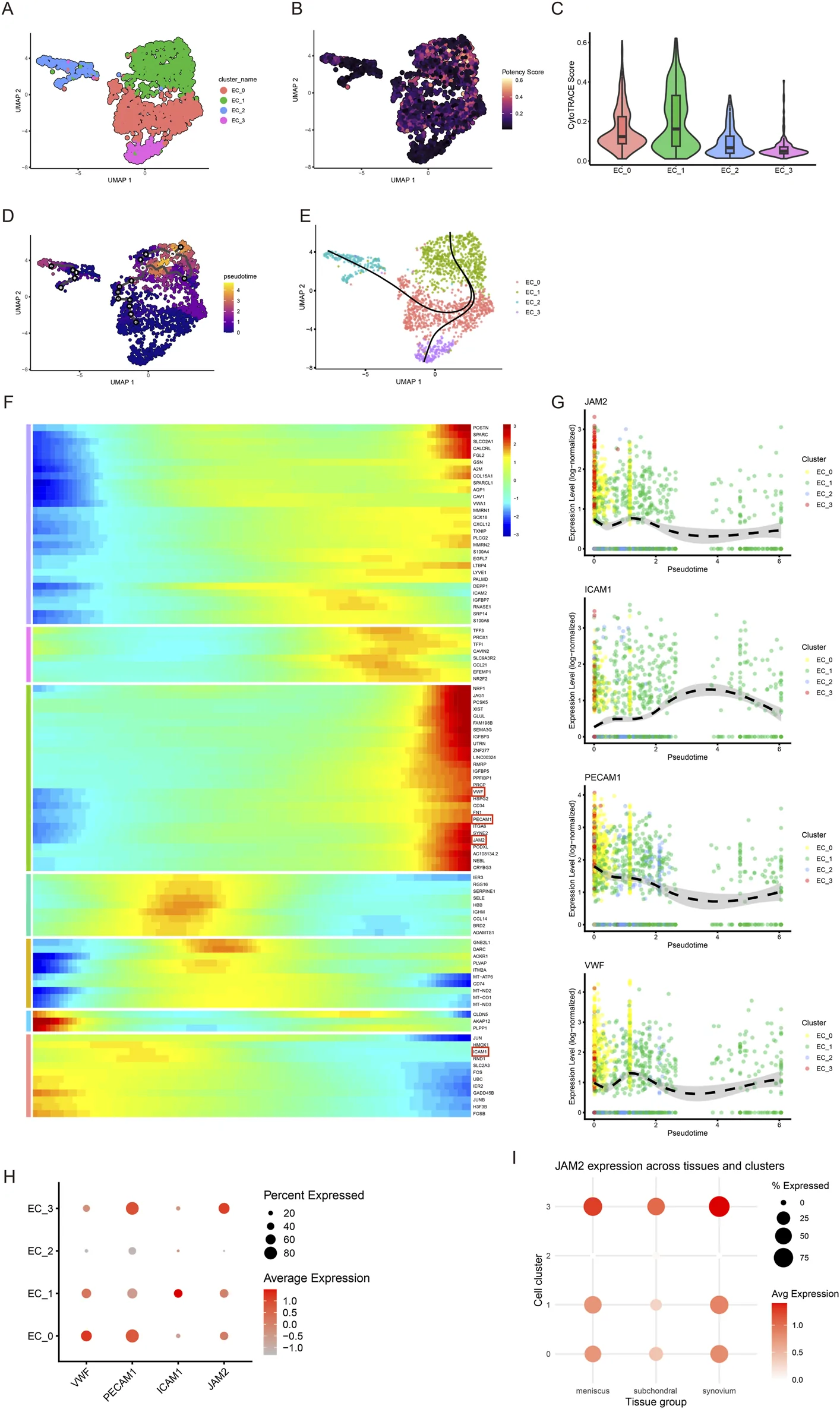
Identification of marker gene of EC_3 and the potential differentiate trajectory. **(A)** UMAP plot showing the four EC subclusters (EC_0, EC_1, EC_2, and EC_3) identified in the scRNA-seq dataset. **(B)** UMAP plot showing the distribution of CytoTRACE scores across the four EC subclusters. **(C)** Violin plot showing the CytoTRACE scores of the four EC subclusters. **(D)** UMAP plot showing the inferred differentiation trajectory of EC subclusters generated by Monocle analysis. **(E)** UMAP plot showing the inferred pseudotemporal trajectory of EC subclusters generated by SingleShot analysis, the black line representing the predicted differentiation trajectory. **(F)** Heatmap showing the top 100 genes related to differentiation. **(G)** Pseudotime kinetics of adhesion related gene and transcript factor in EC. **(H)** Dot plot showing the expression of JAM2 across the four EC subclusters. **(I)** Dot plot showing JAM2 expression across EC subclusters in different tissue types.

Consistent with these findings, trajectory analyses using Monocle and SingleShot supported a potential differentiation trajectory in which EC_1 transitions toward EC_0 and subsequently differentiates into either EC_2 or EC_3 (Figures 5D,E). Although the distribution of endothelial cells and inferred pseudotime values showed some variation across different tissue sources (Supplementary Figures S1A,D), cell-cycle states (Supplementary Figures S1B,E), and individual samples (Supplementary Figures S1C,F), these factors collectively accounted for only 8.5% of the total variance (Supplementary Figure S1G). The remaining 91.5% of the variance was not explained by these factors, supporting the robustness of the inferred differentiation trajectory and suggesting that it was not predominantly driven by tissue source, cell-cycle state, or sample-specific variation.

Together, these analyses suggest a potential developmental trajectory from EC_1 to EC_0, followed by divergence toward EC_2 and EC_3. We next identified the top 100 genes associated with differentiation and systematically evaluated membrane markers to identify a enriched marker for EC_3. Among adhesion-related molecules, we found that JAM2 was differentially expressed in EC_3 compared to other subclusters (Figures 5F,G). The dot plot further confirmed that JAM2 expression was predominantly enriched in EC_3 (Figure 5H). Notably, the elevated expression of JAM2 in EC_3 was consistently observed across different tissue types, with no apparent tissue-specific pattern (Figure 5I). Together, these findings suggest that JAM2 may serve as a potential marker of the EC_3.

### TNF-α upregulates JAM2 in ECs and drives ECs dysfunction

To identify potential upstream regulators of JAM2 in ECs, we selected TNF-α, IL-1β, IL-6 and TGF-β, which represent major inflammatory and tissue-remodeling signals implicated in OA pathogenesis (Kapoor et al., 2011; Cilek et al., 2022). Compared with receptors for IL-6, IL-1β, and TGF-β, TNF-α receptors were highly expressed in ECs (Figure 6A). Subcluster analysis further showed that TNF-α receptors were expressed in EC_0, EC_1, and EC_3 (Figure 6B). Notably, GSEA revealed that TNF-α signaling was the most significantly differentially enriched pathway, with particularly high activity in EC_3 compared with the other EC subclusters (Figure 6C), suggesting that EC_3 may be especially responsive to TNF-α stimulation. To further determine whether TNF-α regulates JAM2 expression in endothelial cells, we treated HUVECs with TNF-α, IL-1β, IL-6, or TGF-β1. Among the cytokines tested, TNF-α markedly increased JAM2 expression at both the mRNA and protein levels (Figures 6D–F). These findings suggest that TNF-α signaling may contribute to the upregulation of JAM2 in EC_3.

**FIGURE 6:**
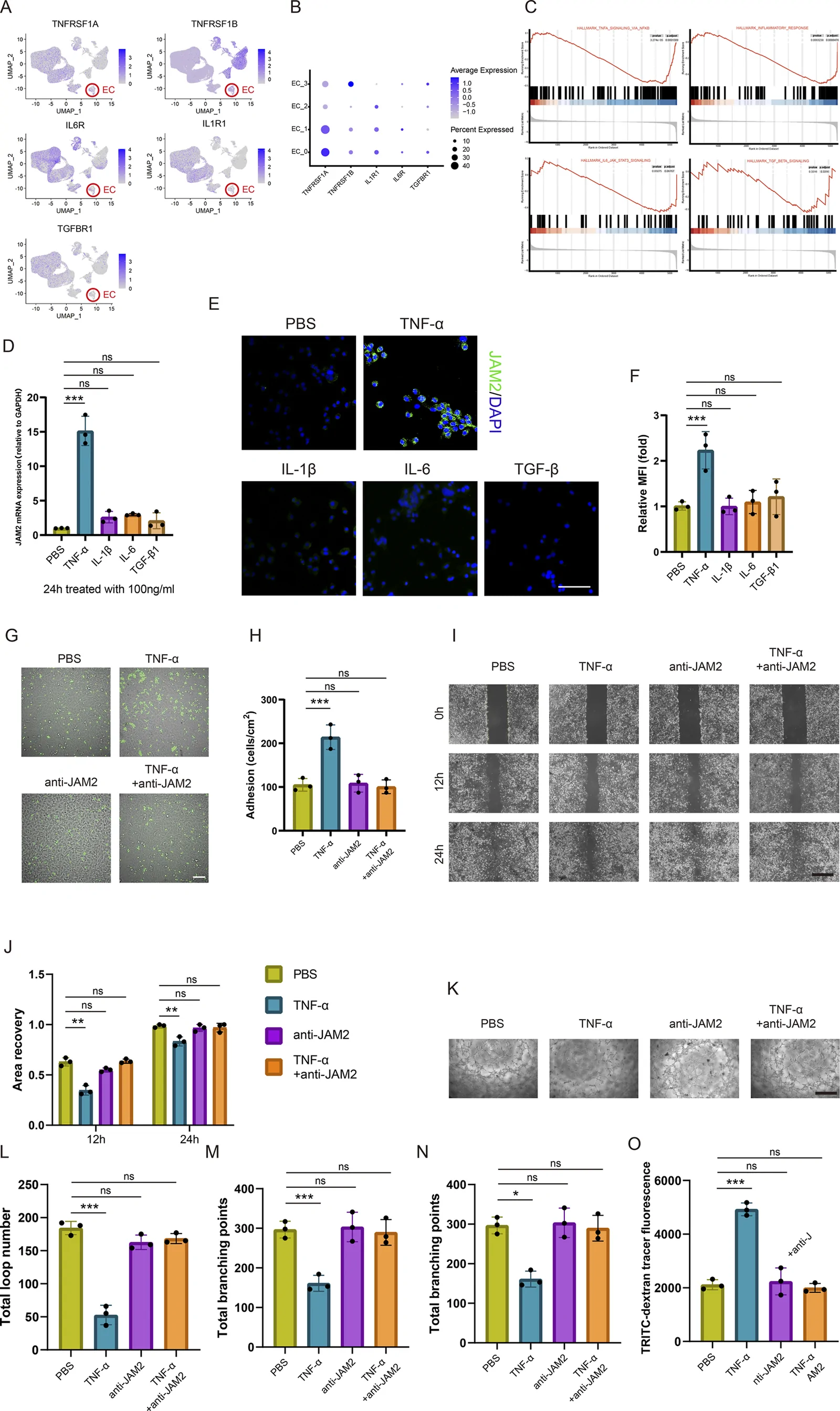
Elevated JAM2 expression in ECs stimulated by TNF-α leads to endothelial dysfunction. **(A)** Feature plots showing the expression of receptors for TNF-α, IL-6, IL-1β, and TGF-β in the scRNA-seq dataset. **(B)** Dot plot showing the expression of TNF-α, IL-6, IL-1β, and TGF-β receptors across the four EC subclusters. **(C)** GSEA plots showing the enrichment of TNF-α, IL-1β, IL-6, and TGF-β signaling pathways in EC_3 compared with the other EC subclusters. **(D)** qRT-PCR analysis of JAM2 mRNA expression in HUVECs treated with TNF-α, IL-1β, IL-6, or TGF-β1 for 24 h (n = 3/group). **(E)** Representative IF images showing JAM2 expression in HUVECs following treatment with TNF-α, IL-1β, IL-6, or TGF-β1 for 24 h. Scare bar, 100 μm. **(F)** Quantification of JAM2 fluorescence intensity in HUVECs. (n = 3/group). **(G)** Representative images showing the adhesion of THP-1 monocytes to HUVECs following different treatments. Scare bar, 100 μm. **(H)** Quantification of adherent THP-1 monocytes on HUVECs. (n = 3/group). **(I)** Representative images of the scratch wound healing assay of HUVECs following different treatments. Scale bar, 200 μm. **(J)** Quantification of area recovery in HUVECs. (n = 3/group). **(K)** Representative images of the tube formation assay in HUVECs following different treatments. Scale bar, 200 μm. **(L)** Quantification of the total number of loops formed by HUVECs in the tube formation assay. (n = 3/group). **(M)** Quantification of the number of branching points in the tube formation assay. (n = 3/group). **(N)** Quantification of the total tube length formed by HUVECs in the tube formation assay. (n = 3/group). **(O)** TRITC-dextran tracer permeability assay showing endothelial permeability of HUVEC monolayers following different treatments. (n = 3/group). *** represents P < 0.001, ** represents P < 0.01, * represents P < 0.05, and ns represents P > 0.05.

To further determine whether TNF-α affects endothelial cell adhesion through upregulation of JAM2, HUVECs pretreated with TNF-α alone or TNF-α in combination with JAM2-neutralizing antibodies were co-cultured with THP-1 monocytes for 30 min. TNF-α stimulation significantly increased the adhesion of THP-1 monocytes to HUVECs, whereas treatment with JAM2-neutralizing antibodies partially attenuated the TNF-α-induced increase in monocyte adhesion (Figures 6G,H). These findings suggest that TNF-α-induced upregulation of JAM2 may contribute to enhanced monocyte–endothelial interactions. We next performed scratch wound healing and tube formation assays to further evaluate the functional effects of JAM2 on endothelial cells. The scratch wound healing assay demonstrated that TNF-α treatment markedly impaired the migratory capacity of HUVECs. Notably, co-treatment with the JAM2-neutralizing antibody partially reversed the inhibitory effect of TNF-α on endothelial cell migration (Figures 6I,J). Consistently, in the tube formation assay, TNF-α-treated HUVECs exhibited a significant reduction in the number of total loops, branching points, and total tube length compared with control cells (Figures 6K–N). Notably, co-treatment with the JAM2-neutralizing antibody partially attenuated the inhibitory effects of TNF-α on the tube-forming capacity of HUVECs. In addition, dextran permeability assays revealed that TNF-α significantly increased endothelial permeability, whereas co-treatment with JAM2-neutralizing antibodies partially reversed the TNF-α-induced increase in endothelial permeability (Figure 6O). Collectively, these findings indicate that TNF-α may promote endothelial dysfunction through the upregulation of JAM2 in endothelial cells.

### JAM2 positive ECs are elevated during osteoarthritis pathogenesis in both humans and mice

To investigate the association between JAM2-positive ECs and OA, we assessed JAM2 expression in the synovium, meniscus, and subchondral bone of OA patients using immunofluorescence. Co-staining with CD31 and JAM2 revealed a significant increase in JAM2-positive ECs in the synovium of OA patients compared with controls (p < 0.05, n = 3 per group) (Figures 7A,B). IgG negative control staining showed no appreciable nonspecific fluorescence, supporting the specificity of the immunofluorescence staining (Supplementary Figure S2).

**FIGURE 7:**
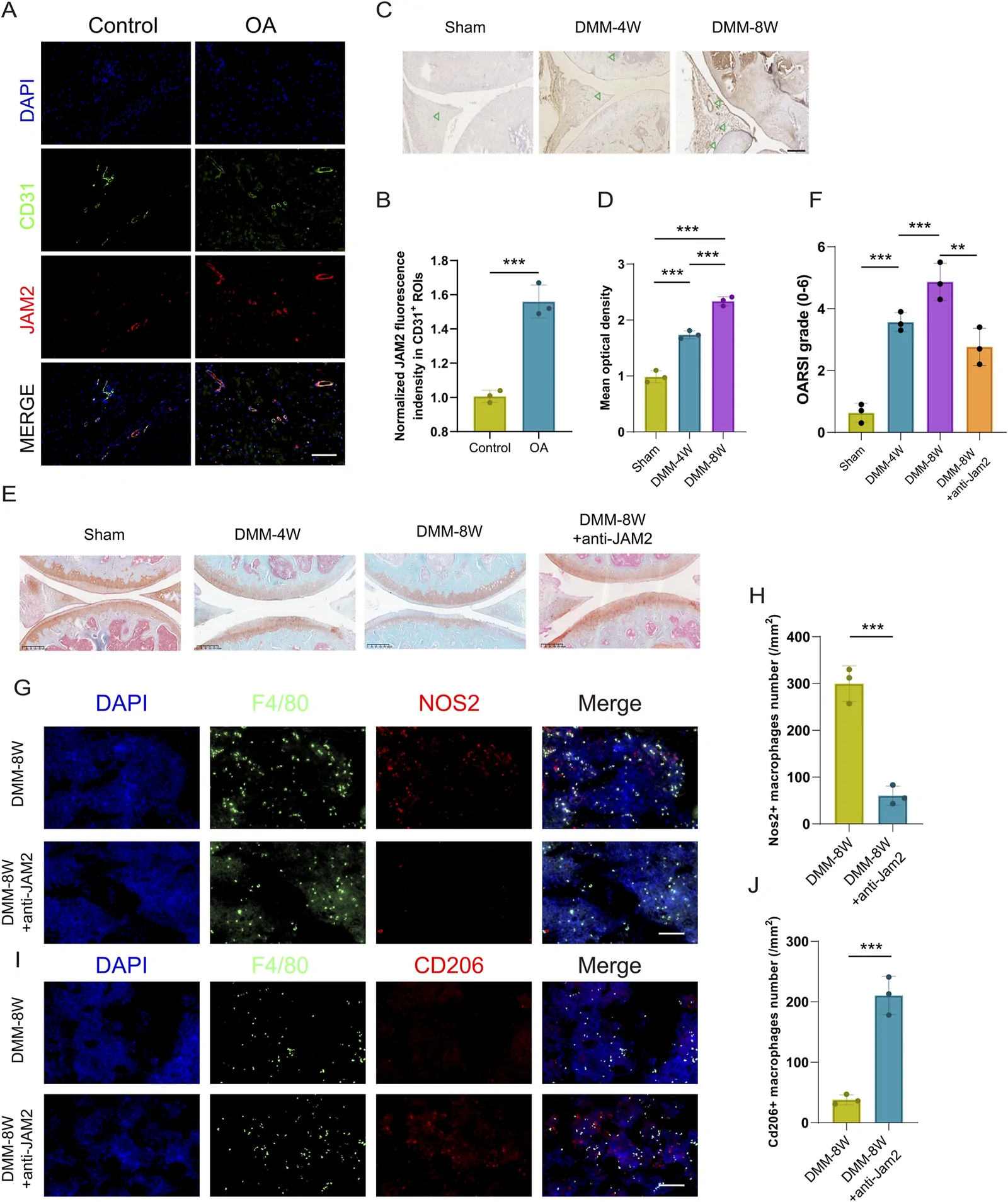
JAM2 expression in ECs are increased during OA progression. **(A)** IF staining of JAM2 in the synovial tissues of healthy controls or patients with knee OA. Scale bar, 100 μm. **(B)** Quantitative analysis of JAM2 expression in the synovial tissues. (n = 3/group). **(C)** IHC staining of JAM2 in the synovium, meniscus, and subchondral bone of sham and DMM mice at 4 and 8 weeks after surgery. Scale bar, 200 μm. **(D)** Quantitative analysis of JAM2 expression of sham and DMM mice at 4 and 8 weeks after surgery. (n = 3/group). **(E)** Representative images of Safranin O/Fast Green staining of knee joint sections from sham mice, DMM mice at 4 and 8 weeks after surgery, and DMM mice treated with JAM2-neutralizing antibodies for 8 weeks after surgery. Scale bar, 200 μm. **(F)** Quantification of cartilage degeneration based on OARSI scores in the indicated groups. (n = 3/group). **(G)** Representative IF images showing F4/80 and NOS2 expression in the synovium of DMM mice treated with JAM2-neutralizing antibodies or control at 8 weeks after surgery. Scale bar, 50 μm. **(H)** Quantification of NOS2+ macrophages in the synovial tissues of the indicated groups. (n = 3/group). **(I)** Representative immunofluorescence images showing F4/80 and CD206 expression in the synovium of DMM mice treated with JAM2-neutralizing antibodies or control at 8 weeks after surgery. Scale bar, 50 μm. **(J)** Quantification of CD206+ macrophages in the synovial tissues of the indicated groups. (n = 3/group). *** represents P < 0.001, ** represents P < 0.01, * represents P < 0.05, and ns represents P > 0.05.

To evaluate JAM2 expression in an animal model of OA, we performed destabilization of the medial meniscus (DMM) surgery in mice and collected knee tissues at 4 and 8 weeks post-surgery. Our IHC analysis showed that JAM2 expression was increased in blood vessels within the meniscus, synovium and subchondral bone with the progression of OA in mice (Figures 7C,D). To evaluate whether JAM2 represents a potential therapeutic target for OA, we performed systemic administration of JAM2-neutralizing antibodies following DMM surgery. Safranin O/Fast Green staining showed that JAM2 blockade attenuated the progression of cartilage damage in DMM mice at 8 weeks after surgery, as evidenced by a significant reduction in OARSI scores (Figures 7E,F). We next investigated whether the therapeutic effects of JAM2 blockade on OA progression were associated with changes in inflammatory cell infiltration. In the synovium of OA mice, treatment with JAM2-neutralizing antibodies significantly reduced the infiltration of M1-like macrophages (Figures 7G,H), while increasing the infiltration of M2-like macrophages (Figures 7I,J). Collectively, these findings suggest that JAM2 blockade may attenuate OA progression by reducing cartilage degeneration and modulating the inflammatory macrophage response in the synovial microenvironment. These findings provide *in vivo* evidence supporting JAM2 as a potential therapeutic target for OA.

## Discussion

Increasing evidence suggests that osteoarthritis (OA) is not merely a result of simple cartilage wear and tear but rather a complex joint disease involving subchondral bone lesions and synovitis, which precede cartilage degeneration in OA (Wei and Bai, 2016; Perry et al., 2020; Hu et al., 2021). During the progression of OA, the increased angiogenesis in the synovium and the vascular invasion from subchondral bone into the cartilage further complicate our understanding of the disease (Xiaoshi et al., 2021). However, the exact role of endothelial cells (ECs) in OA initiation and progression, including their interactions with immune cells and other joint tissues, remains unclear. Therefore, it is essential to uncover the role of ECs in OA and identify key subclusters involved in this crosstalk. In this study, we employed comprehensive single-cell RNA sequencing (scRNA-Seq) to elucidate the diverse cell types in the knee OA microenvironment.

We identified 13 major cell clusters in the human knee OA microenvironment, including endothelial cells (ECs), pericyte-like cells, macrophages, mast cells, and T cells, which were present across the subchondral bone, meniscus, and synovium. This broad distribution highlights the importance of vascular and immune cell involvement in OA pathogenesis. To validate the significance of ECs in this environment, we isolated and further analyzed ECs from the 13 identified clusters to assess their heterogeneity and regulatory functions.

Previous studies have shown that the endothelial barrier plays a crucial role in maintaining tissue homeostasis by regulating the exchange between local tissues and circulating cells (Chen et al., 2021). In OA, avascular cartilage undergoes degenerative changes, often accompanied by neovascularization (Ruscitto et al., 2020). Meanwhile, the synovium, subchondral bone, and the red zone of the meniscus frequently undergo inflammatory changes and immune cell infiltration (Tuckermann and Adams, 2021). In these contexts, ECs play a pivotal role in cellular exchange and immune modulation. Several mechanisms through which blood vessels participate in OA development have been proposed. For instance, Findlay et al. suggested that episodic reductions in blood flow through small vessels of the subchondral bone due to vascular pathology contribute to OA initiation and progression (Muratovic et al., 2022). Studies have also demonstrated a correlation between vascular endothelial growth factor (VEGF) expression and OA severity in both humans and mice (Nagao et al., 2017; Takano et al., 2018). Additionally, Bevacizumab, a VEGF inhibitor, has been shown to suppress degenerative changes in articular cartilage (Nagai et al., 2014; Sotozawa et al., 2023).

Research by Yang et al. indicates that exosomes secreted by vascular endothelial cells can contribute to OA pathogenesis by promoting chondrocyte apoptosis (Yang et al., 2021). More recently, type H vessels have been identified as critical players in connecting subchondral bone remodeling with articular cartilage degeneration in OA development (Kusumbe et al., 2014; Peng et al., 2020). Blocking the formation of these H-type vessels or disrupting their positive feedback regulation can attenuate OA in animal models (Cui et al., 2016). These findings underscore the importance of studying EC subtypes in OA. However, technical limitations and the insufficient quantity of ECs have previously hindered our understanding of the vascular pathology and heterogeneity in joint diseases.

Using scRNA-Seq, we created a single-cell transcriptome atlas of ECs in OA and identified four distinct EC subclusters (EC_0, EC_1, EC_2, and EC_3). We further characterized the molecular and functional perturbations in knee OA vessels. One of the major features of endothelial dysfunction in OA is increased vascular permeability and reduced barrier function, leading to enhanced immune cell adhesion and transmigration (Kumar et al., 2021). Interestingly, we found that EC_3, which is enriched in genes related to vessel morphogenesis, focal adhesion, and leukocyte transendothelial migration, exhibited significantly higher interactions with other cell clusters. Using the CytoTRACE method to determine the pseudotemporal order between EC subclusters and Monocle2 to identify differentiation-related genes, we identified JAM2 as a membrane marker specific to EC_3 (Aurrand-Lions et al., 2001a; Aurrand-Lions et al., 2001b). Notably, JAM2 was highly expressed in EC_3, and we hypothesize that JAM2-positive ECs actively respond to OA cytokine signaling, preparing the endothelium for immune cell adhesion and transmigration.

Our functional enrichment analysis and *in vitro* experiments confirm that TNF-α stimulation increases JAM2 expression in ECs, impairing tube formation and cell migration. Moreover, ECs stimulated with TNF-α exhibited increased adhesion of THP-1 cells, suggesting that EC_3 plays a role in recruiting immune cells and regulating their extravasation during OA progression. These findings highlight JAM2-positive ECs as a specialized subset of ECs in OA that may facilitate immune cell trafficking and contribute to OA inflammation.

Junctional adhesion molecules (JAMs) are essential regulators of endothelial and epithelial permeability, leukocyte recruitment during inflammation, angiogenesis, and cell migration (Ebnet et al., 2003). JAMs are broadly distributed across various tissues and cell types (Bazzoni, 2003). Due to their presence in both ECs and leukocytes, members of the JAMs family have been extensively studied for their roles in vascular homeostasis and leukocyte trafficking (Arcangeli et al., 2013; Gerhardt and Ley, 2015). Among the JAMs family, JAM2 has a more restricted distribution, particularly localized at the tight junctions of ECs. It is predominantly found at the junctions of endothelial cells in high endothelial venules, which are responsible for regulating the entry of blood-borne lymphocytes into lymphatic tissues in response to processed antigens (Aurrand-Lions et al., 2001a; Johnson-Leger et al., 2002). Previous studies have shown that JAM2 in ECs facilitates T cell extravasation (Johnson-Leger et al., 2002; Liang et al., 2002).

In the context of OA, we found that JAM2-positive ECs are activated via the TNF-α signaling pathway. Upon TNF-α stimulation, there was a significant increase in JAM2 expression in ECs, accompanied by a notable reduction in angiogenesis and enhanced adhesion of THP-1 cells. Based on Gene Ontology (GO) analysis and *in vitro* experiments, our results suggest that JAM2-positive ECs exhibit a heightened ability to recruit immune cells and a diminished capacity for angiogenesis. These findings support the hypothesis that JAM2-positive ECs play a central role in the inflammatory response in OA by promoting immune cell adhesion and transendothelial migration, while simultaneously impairing vascular repair mechanisms.

Our *in vivo* data further highlight the importance of JAM2-positive ECs in OA pathogenesis. In OA patients, we observed an expansion of JAM2-positive ECs in the synovium compared to healthy controls. Additionally, in a mouse model of OA induced by destabilization of the medial meniscus (DMM), JAM2 expression progressively increased in the subchondral bone over time. These findings indicate that the proliferation of JAM2-positive ECs correlates with OA progression. Similarly, a study by Muller (2003) demonstrated that blocking JAM2- JAM3 interactions reduces monocyte infiltration in extravascular tissues by promoting reverse transmigration. Our results expand on these observations, revealing that upregulation of JAM2 expression in ECs at OA sites contributes to the increased presence of JAM2-positive ECs, which may further exacerbate OA inflammation by facilitating immune cell adhesion. Importantly, therapeutic administration of JAM2-neutralizing antibodies significantly attenuated OA progression in DMM mice. These findings suggest that JAM2 may represent a potential therapeutic target for OA.

Previous research suggests that inhibition of proinflammatory cytokines, such as TNF-α, and the reduction of leukocyte adhesion to ECs, are critical strategies for managing joint inflammatory diseases (Voirin et al., 2020; Liu et al., 2019). Based on our findings, we propose that TNF-α stimulation upregulates JAM2 expression in ECs, which in turn enhances the adhesion of inflammatory cells, such as macrophages, to the endothelium. Importantly, treatment with JAM2-neutralizing antibodies partially attenuated the TNF-α-induced increase in inflammatory cell adhesion to ECs, further supporting a role for JAM2 in mediating this process. This process likely exacerbates the progression of OA by promoting immune cell infiltration and sustaining local inflammation. Therefore, targeting JAM2 expression in endothelial cells and inhibiting immune cell adhesion could provide novel therapeutic approaches for treating OA.

While our study underscores the importance of JAM2-positive ECs in regulating immune cell adhesion and contributing to OA progression, several limitations should be acknowledged. First, although our findings indicate that TNF-α promotes JAM2 expression in endothelial cells, the specific molecular mechanisms underlying TNF-α-induced JAM2 upregulation remain unclear. Second, although JAM2-neutralizing antibodies treatment provided functional evidence that JAM2 contributes to TNF-α-induced endothelial dysfunction, the current study did not include genetic loss-of-function approaches, such as JAM2 knockdown or CRISPR interference, or formal rescue experiments. In addition, the effects of JAM2 blockade on endothelial inflammatory gene expression were not directly evaluated. Third, although our *in vitro* and *in vivo* experiments provide evidence supporting a role for JAM2-positive ECs in endothelial dysfunction and immune cell interactions, conventional cell culture systems and animal models may not fully recapitulate the complex multicellular and tissue-specific microenvironment of human OA. In this regard, organoid models may provide a more physiologically relevant platform for investigating the interactions among endothelial cells, immune cells, and other joint-resident cell populations in a three-dimensional microenvironment (Gao et al., 2025). Future studies using OA organoid models incorporating JAM2-positive ECs will be valuable for determining how TNF-α–JAM2 signaling regulates endothelial–immune cell interactions and for evaluating the therapeutic potential of targeting this axis in a more clinically relevant setting. Fourth, the relatively small number of animals included in the current analysis is another limitation of this study. The limited sample size may reduce the robustness and generalizability of the *in vivo* findings, and further studies using larger and independently replicated animal cohorts are warranted to validate our conclusions. Overall, our findings identify JAM2-positive ECs as important contributors to OA progression and provide a basis for further investigation of the TNF-α–JAM2 axis in OA.

## Conclusion

In conclusion, to our knowledge, this study is the first to identify JAM2-positive inflammation-associated ECs in OA progression. We demonstrate that JAM2-positive ECs facilitate immune cell adhesion and transmigration, correlating with OA severity. These findings highlight the importance of JAM2 in endothelial dysfunction during OA and suggest that targeting JAM2-positive ECs could represent a novel therapeutic strategy to mitigate inflammation and slow the progression of OA. Further studies are needed to explore the precise mechanisms by which JAM2 contributes to OA pathogenesis and to evaluate potential therapeutic interventions targeting this pathway. Ultimately, our work provides new insights into the endothelial dysfunction and immune cell interactions that drive OA progression, paving the way for the development of targeted therapies for this debilitating disease.

## Data Availability

The datasets presented in this study can be found in online repositories. The names of the repository/repositories and accession number(s) can be found in the article/Supplementary Material.

## Supplementary material

SUPPLEMENTARY FIGURE S1 Evaluation of potential confounding factors in the inferred endothelial cell trajectory. **(A)** UMAP visualization of endothelial cell subclusters colored according to tissue source. **(B)** UMAP visualization of endothelial cell subclusters colored according to cell-cycle state. **(C)** UMAP visualization of endothelial cell subclusters colored according to individual sample. **(D)** Violin plot showing the distribution of inferred pseudotime values across different tissue sources. **(E)** Violin plot showing the distribution of inferred pseudotime values across different cell-cycle states. **(F)** Box plots showing the distribution of inferred pseudotime values across individual samples. **(G)** Stacked bar plot showing the relative contributions of tissue source, cell-cycle state, and individual sample to the total variance in the inferred trajectory.

SUPPLEMENTARY FIGURE S2 IgG negative control staining for JAM2 and CD31 immunofluorescence. Representative IF images showing IgG negative controls for JAM2 and CD31 staining in human synovial tissue. Scale bar, 200 μm.
