# Supplementary material for: Single cell RNA-seq analysis identifies junctional adhesion molecule two positive endothelial cells aggravate osteoarthritis by mediating vascular permeability and macrophage adhesion
Source: Front Cell Dev Biol. 2026 Oct 9;14:1937741. doi: 10.3389/fcell.2026.1937741 (PMC13690561; doi:10.3389/fcell.2026.1937741)
Supplement: Supplementary file 1 [file Table1.docx]

**Supplementary Table S1. Baseline characteristics of human study participants**

| Patient ID | Disease | Age (years) | Sex | Side | BMI (kg/m²) | Surgical procedure | Synovial tissue source |
| --- | --- | --- | --- | --- | --- | --- | --- |
| OA-1 | Osteoarthritis | 67 | Male | Left | 27.3 | TKA | Knee synovium |
| OA-2 | Osteoarthritis | 59 | Female | Left | 26.5 | TKA | Knee synovium |
| OA-3 | Osteoarthritis | 63 | Male | Right | 28.7 | TKA | Knee synovium |
| OS-1 | Osteosarcoma | 11 | Female | Left | 16.7 | Amputation | Knee synovium |
| OS-2 | Osteosarcoma | 14 | Female | Right | 13.2 | Amputation | Knee synovium |
| OS-3 | Osteosarcoma | 13 | Male | Right | 15.6 | Amputation | Knee synovium |
